# Exploitation of the Mediator complex by viruses

**DOI:** 10.1371/journal.ppat.1010422

**Published:** 2022-04-21

**Authors:** Joel Rovnak, Sandra L. Quackenbush

**Affiliations:** Department of Microbiology, Immunology and Pathology, Colorado State University, Fort Collins, Colorado, United State of America; University of Iowa, UNITED STATES

## Viruses and the discovery of Mediator

Just as biophysicists were identifying the component proteins of functional RNA polymerase II (RNAPII) complexes, virologists were identifying viral proteins that control it with peculiar efficiency [1]. Purified transcription components phosphorylate the RNAPII carboxyl-terminal domain (CTD) to initiate transcription, and this is enhanced by viral transactivators. In this way, viral proteins with their potent transcription activation domains (TADs) served as powerful tools for the characterization of DNA binding domains and TAD structures, e.g., the “acidic activator” (an acidic amphipathic alpha helix) and in the identification of host proteins that control transcription. The herpes simplex virus (HSV) transactivator, virion protein 16 (VP16, α-TIF, and Vmw65), was particularly valuable in transcription assays [1–3].

In the early 90s, prior to discovery of Mediator, a cyclin of unknown function, cyclin C, identified in screens for the rescue of yeast deficient in *CLN* genes (G1 cyclins) [4–6], was found to activate cyclin-dependent kinase 8 (CDK8) to phosphorylate the RNAPII CTD, placing it firmly at transcription control, not cell cycle [7–9]. At the same time, virologists discovered that VP16 and the adenovirus E1A proteins were associated with a large complex with cyclin C/CDK8 kinase activity [10].

This same period saw the sequence of an acutely transforming oncogenic retrovirus, walleye dermal sarcoma virus (WDSV) [11]. The WDSV genome encodes a retroviral cyclin (RV-cyclin) with only distant homology to any eukaryotic cyclin. The RV-cyclin rescued *CLN*-deficient yeast and induced hyperplastic lesions in mice carrying its transgene [12,13].

At this point, many components of Mediator had been identified as parts of a large, thyroid-hormone receptor complex (TRAP, THRAP, activator-recruited cofactor (ARC), and cofactor required for Sp1 activation (CRSP)) [14,15], and in 2002, the whole ARC/CRSP complex (Mediator) was purified by its VP16 affinity and its structure visualized by cryo-EM [16]. We showed that the RV-cyclin colocalized with transcription and splicing complexes in nuclei of mammalian cells and, in 2002, demonstrated its specific binding to and activation of human CDK8 (walleye and human CDK8 proteins are 98% identical) [17,18]. This was the first association of CDK8 with oncogenesis. In 2008, CDK8 was identified as an oncogene in a majority of human colon carcinomas and has since been implicated in a variety of human metastatic cancers [19,20].

The evolution of a viral cyclin that binds and activates the CDK component of Mediator illustrates the value of Mediator control for virus replication. While RV-cyclin is the only virus protein known to directly activate Mediator CDKs, the TADs of many viral transactivators function via direct contact with Mediator proteins, and additional virus protein contacts are being identified. Mediator offers refined control of host and virus gene expression, and viruses avail themselves of this control to meet their specific needs.

## What is the Mediator complex?

RNAPII transcription is initiated by transcription factors bound to enhancer and promoter regions, recruitment of coactivator complexes that modify and remodel chromatin, and assembly of preinitiation complexes (PICs) with general transcription factors and RNAPII at core promoters. The Mediator complex, does just what it was named for: It mediates signals from transcription factors to assembled RNAPII complexes. Mediator is a bridge between a diverse array of DNA recognition factors and RNAPII. It also positions CDK7 at the RNAPII CTD for phosphorylation and transcription initiation [21–23]. Mediator coordinates and refines outputs from an array of disparate signals by reconfiguration of its components to control transcription initiation, elongation, and termination [24–26]. It is a cofactor in all RNAPII transcription [27,28].

Mediator is composed of approximately 30 proteins in 4 structural modules: the head, middle, and tail modules comprise the core, which can associate with the CDK8 kinase module (CKM) [29]. The Mediator core stabilizes the association of CDK7 in the general transcription factor TFIIH with the RNAPII CTD for phosphorylation of serine 5 in the CTD heptad repeats to initiate transcription [21–23,29–31]. The CKM is dissociable from Mediator. Its presence in the complex inhibits transcription initiation by blocking CDK7 access to the RNAPII CTD. However, the CKM is necessary for the release of paused polymerase complexes in response to specific enhancer-bound transcription factors that reprogram gene expression [29,32,33].

The CKM includes the CDK8-activating cyclin, cyclin C, and Mediator proteins, Med12 and Med13. MED12 increases CDK8 activation, and MED13 links the CKM with the core [31,34,35]. A CDK8 paralog, CDK19, present only in vertebrates, shares the kinase domain of CDK8 but diverges in the carboxyl-terminal tail. The CDK19 module includes cyclin C and Med12L and Med13L. CDK8 and CDK19 are highly conserved serine–threonine kinases that phosphorylate serines 2 and 5 in the heptad repeats of the RNAPII CTD to control transcription pausing and elongation, chromatin remodeling, and RNA processing and export [36–39]. CDK8 also phosphorylates itself, cyclin C, Med12 and Med13, Mediator middle and head modules, Cyclin H, multiple transcription factors, general transcription factors, the super elongation complex (SEC), chromatin remodelers, and serine 10 of histone H3 (H3S10) [8,9,40–43]. Autophosphorylation of the CKM yields full activation and controls CKM–Mediator association and Med13 degradation. Phosphorylation of Mediator middle and head modules controls the structure of core Mediator. Phosphorylation of Cyclin H, the CDK7 activator, inhibits transcription initiation. Phosphorylation of transcription factors controls reprogramming of gene expression, especially in response to metabolic, proliferative, and developmental signals by altering their activity or stability. Physical interactions between CKM–Mediator and pTEFb/SEC complexes at paused polymerases and SEC phosphorylation support pause release [44]. H3S10 phosphorylation by dissociated CKM promotes H3K9 demethylation and H3K14 acetylation and transcription activation [43].

CDK8 activity regulates nutrient stress response genes in yeast [45,46]. In metazoan organisms, CDK8 activity is necessary to reprogram cell identity and proliferation in response to stimuli like cytokines and serum and metabolism in response to cell stresses, such as hypoxia and starvation [36,37,42,47–50]. CDK8 controls transcription responses to Ras/Mitogen-activated protein (MAPK), Wingless/Integrated (WNT), transforming growth factor beta (TGFβ), nuclear factor kappa B (NF-κB), interferon gamma (IFNγ), and Janus kinase (JAK)/signal transducer and activator of transcription (STAT) pathways [42,51–53]. The CKM controls responses to a wide array of internal and external signals but has little control of steady-state house-keeping genes [32]. A possible selective mechanism for Mediator CKM dependent gene expression programs lies in the differential recruitment of elongation factors by the CKM versus Med26 of CKM-free Mediator complexes [29].

Ectopic RV-cyclin increases CDK8 kinase activity for histone H3 and RNAPII CTD in vitro. It also increases and extends the duration of CDK8 and RNAPII occupancy across the loci of serum response genes, prior to and after stimulation. This increases total mRNA levels from these CDK8-dependent genes and significantly increases cell proliferation [18,54–56].

## Viruses target Mediator to activate viral gene expression

**Fig 1:**
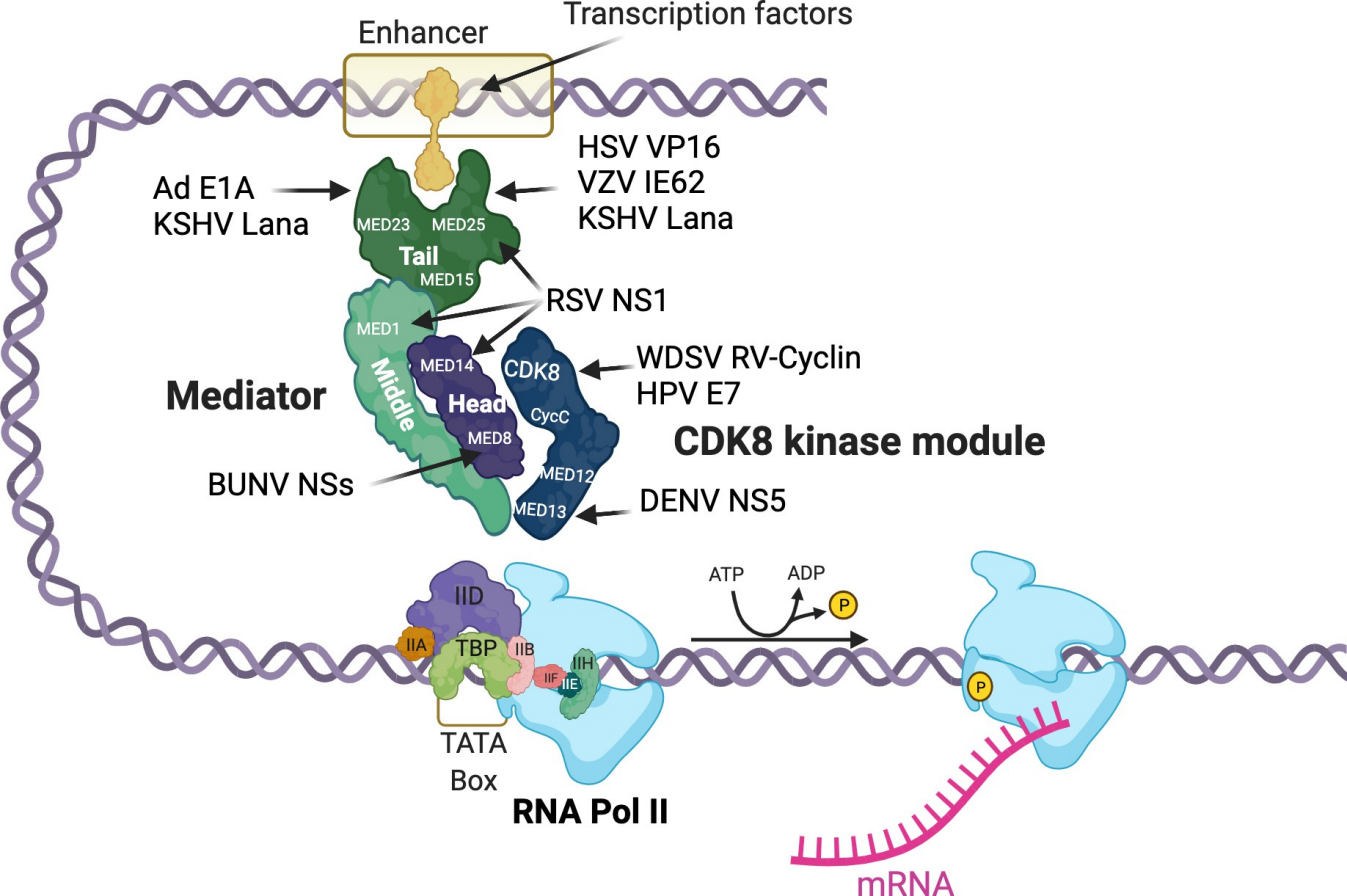
Viral proteins target the Mediator complex to control viral and host gene expression. Viral proteins known to bind to or associate with Mediator proteins in the tail, middle, and head and CDK modules are depicted. Ad, adenovirus E1A; BUNV, Bunyamwera virus NSs; CDK8, cyclin-dependent kinase 8; DENV, dengue virus NS5; HPV, human papilloma virus E7; HSV, herpes simplex virus VP16; KSHV, Kaposi sarcoma–associated herpesvirus Lana-1; RSV, respiratory syncytial virus NS1; RV-cyclin, retroviral cyclin; VZV, varicella zoster virus IE62; WDSV, Walleye dermal sarcoma virus. Created with BioRender.com.

DNA viruses and retroviruses depend on host transcription machinery and encode proteins that control the extent and timing of their gene expression. This allows coordination of virus replication with capsid construction and packaging. The TADs of viral factors make high affinity contacts with Mediator proteins to activate and maintain high transcription rates of virus genes (Fig 1). These viral proteins served as models of host factors and led to the identification of specific Mediator protein structures, such as ACID, the activator interaction domain, in Med25, named for the VP16 acidic activator [57,58]. Other examples include the Kaposi’s sarcoma–associated herpesvirus Lana-1 protein, which binds Med15, Med23, and Med25 [59], the Varicella Zoster Virus IE62 protein, which targets Med25 [60], and the adenovirus E1A protein, which binds Med23 (SUR-2) [61]. For human papilloma virus 16 (HPV16), increased CDK8 occupancy on the long control region enhancer and late promoter in differentiating cells is associated with late promoter activity [62]. The WDSV RV-cyclin actually inhibits expression from the virus promoter during the latent phase of infection but carries a potent acidic TAD that activates host proliferative genes [63].

## Viruses target Mediator to control cell differentiation and proliferation

Many viruses require host machinery to replicate their genome. For small DNA tumor viruses, polyoma, papilloma, and adenoviruses, host–cell proliferation provides DNA polymerase components that replicate viral DNA. Proteins encoded by these viruses target pRb and p53 to trigger cell cycle and block apoptosis and may also contact Mediator to control host gene expression, e.g., E1A [61].

The lymphotropic retroviruses and herpesviruses persist as integrated genomes or tethered episomes, so host cell proliferation replicates the viral genome. These viruses encode proteins that manipulate host transcription factors that contact Mediator tail proteins to promote cell division. Transition to virus production is also maintained via tight control of host transcription and requires Mediator.

A refined interplay between host proliferative response and virus latency and lytic replication is modeled in the seasonal development and regression of WDSV. Latent provirus produces low levels of RV-cyclin and OrfB transcripts. OrfB protein constitutively activates protein kinase C and AKT [64] to activate host immediate early genes, *FOS*, *JUN*, and *EGR1*. RV-cyclin increases CDK8 activity and chromatin occupancy of these genes to increase their rates of transcription elongation and reinititation [54]. After a season of tumor cell and provirus proliferation, the WDSV expression profile suddenly switches to genome replication, particle formation, and tumor regression timed with host spawning. Ectopic RV-cyclin alone can increase division even of highly proliferative human cancer cells [55]. This model ties CDK8 activity and chromatin occupancy to transcription elongation and reinitiation and to cell proliferation [54].

## Viruses target Mediator for control of immune response

Viruses evolved many mechanisms to antagonize innate and adaptive immunity, especially interferon response pathways. Virus proteins that target transcription of key antiviral genes include those that contact Mediator. CDK8 and CDK19 regulate transcriptional responses to IFNγ [42,65], and CDK8 promotes release of paused RNAPII at IFNγ-induced genes [65]. Knockdown of cyclin C or CDK19 makes cells more sensitive to virus infection [42,65], indicating their elemental control of antiviral responses.

Orthobunyaviruses encode a nonstructural protein, NSs, which antagonizes the type I interferon response. Bunyamwera NSs binds Med8 in the head domain of Mediator to prevent phosphorylation of serine 2 in the RNAPII CTD and inhibit elongation of mRNA [66,67]. Human respiratory syncytial virus NS1, another antagonist of interferon signaling, contacts Med1, Med14, and Med25 and is enriched at enhancer regions, including enhancers of interferon-stimulated genes (ISGs), suggesting a role in their modulation [68]. HPV16 E7 also suppresses ISG transcription via CDK8, and knockdown of CDK8 in E6/E7-expressing cells increases ISG expression [69].

## Viruses target Mediator for control of host cell metabolism

Virus infection frequently results in reprogramming of host cell metabolism in ways that support virus replication. As indicated above, Mediator, and CDK8 in particular, are key regulators of metabolic reprogramming. Dengue virus enhancement of glucose metabolism and oxidative phosphorylation is well characterized [70–72]. Infection induces CDK8 expression, and CDK8 activity is required for the up-regulation of metabolic genes [73]. The dengue virus RNA-dependent RNA polymerase, NS5, has high affinity for host chromatin and associates with Med13 [73,74] (personal communication, R. Perera). NS5 also interacts with spliceosomes to dysregulate splicing to favor virus replication and with polymerase associated factor 1 complex (PAF1C) to antagonize expression of PAF1-dependent immune response genes [75,76].

The question remains as to a specific role for NS5 and similar viral proteins in the direct control of host gene expression. There are many cell signaling pathways, triggered by virus infection and replication, which would result in metabolic reprogramming without benefit of direct virus control of Mediator. However, as we move into a new era of Mediator control of nuclear condensates to stabilize transcription and splicing components in response to cell signaling [52], we may reconsider the roles of viral proteins such as NS5 that have high concentrations on active chromatin [73,74]. Whether they guide or enhance reprogramming, viral proteins will once again serve as models for for investigations of eukaryotic gene expression.
